# Factors associated with fetal macrosomia

**DOI:** 10.11606/s1518-8787.2019053001269

**Published:** 2019-11-18

**Authors:** Vanessa Agudelo-Espitia, Beatriz Elena Parra-Sosa, Sandra L Restrepo-Mesa

**Affiliations:** I Universidad de Antioquia. Escuela de Nutricion y Dietetica. Colombia; II Universidad de Antioquia. Escuela de Nutrición y Dietética. Grupo de Investigación Alimentación y Nutrición Humana. Magíster en Ciencias Básicas Biomédicas. Colombia; III Universidad de Antioquia. Escuela de Nutrición y Dietética. Grupo de Investigación Alimentación y Nutrición Humana. Magíster en Salud Colectiva. Colombia

**Keywords:** Fetal Macrosomia, Risk Factors, Birth Weight, Body Weight Gain, Prenatal Nutritional Physiological Phenomena, Maternal and Child Health

## Abstract

**OBJECTIVE:**

To evaluate the clinical factors, as well as weight gain, in a group of pregnant women, associating them with fetal macrosomia in a public institution in Antioquia, Colombia, from 2010-2017.

**METHODS:**

A case-control study, using secondary information registries. Cases were defined using newborn weight of ≥ 4000g, while controls were defined as newborn weight between 3000–3999g. A proportion ratio (PR) was established to evaluate factors associated with macrosomia, and a generalized linear model (GLM) of Poisson regression with robust variance was used to evaluate the aspects that best explained macrosomia in the neonate.

**RESULTS:**

122 pregnant women participated in the study, of which 611 were cases and 61 were controls. Of the participants, 44.3% had pre-pregnancy overweight and 48.4% had excess gestational weight gain. Statistically significant differences were found between the groups in the following variables: pre-pregnancy BMI (p = 0.004), gestational weight gain (p = 0.000), gestational diabetes (p = 0.000), and type of delivery (p = 0.004). According to the regression model, a macrosomic newborn is 3.5 times more likely in women with excessive gestational weight gain (95%CI 1.78-7.18) and twice more likely in women who have gestational diabetes (95%CI 1.51-2.76). Of women with pre-pregnancy excess weight, 63% had excess gestational weight gain.

**CONCLUSIONS:**

Within this cohort, pre-pregnancy BMI, excess weight gain in pregnancy, and the presence of gestational diabetes were associated with an increased risk of neonatal macrosomia. pre-pregnancy BMI and weight gain in pregnancy are modifiable risk factors that are responsive to nutrition interventions, which can minimize adverse perinatal outcomes.

## INTRODUCTION

The figures of excess weight have had an increase in the world population in recent decades. Report of the Panorama of Food and Nutrition Security in Latin America and the Caribbean has shown that overweight and obesity, in more than 20 countries on the continent, was 10 percentage points higher in women than in men in 2016^1^ . A report prepared in 2015 by the United Nations states that one in four women in adulthood is obese^2^ . The situation is no different in Colombia: the prevalence of overweight and obesity in the adult population is 55.2% in women compared with 45.6% in men^3^ .

More and more women begin pregnancy in excess weight. The 2015 National Survey of Nutrition Situation in Colombia indicates that 39.9% of pregnant women of all age groups were overweight (24.7% overweight and 15.2% obese) in the country^3^ . Other observational studies in representative samples of pregnant women in countries such as Peru^4^ , Brazil^5^ , and Uruguay^6^ show overweight prevalence of 63.8%, 47.5% and 32.6%, respectively.

This scenario directly affects birth weight. A study in 23 countries showed a prevalence of macrosomia of 4.5% and 5.4% in Latin America. In developed countries, it ranged between 5% and 20%, and a 15-25% increase has been reported in the last three decades^7^ . In Colombia, an investigation was conducted based on the Live Birth Registry of the Administrative Department of Vital Statistics from 2002 to 2011, among which about 6,000,000 births were registered. Low birth weight amounted to 3.8%, while macrosomia reached 4.5% in full-term newborns (NB)^8^ .

The excess weight-gestation binomial can mark the origin of a range of diseases. Various mechanisms seem to come together in the metabolic programming and generational transfer of obesity and its associated comorbidities: inflammation, oxidative stress, neurohormonal disorders, epigenetic modifications, quality of the maternal microbiota, macrosomia and greater fetal adiposity^9^ ; in addition to greater admission to the neonatal intensive care unit, respiratory disturbances and neonatal death^10^ .

Risks of excess weight in the mother include preeclampsia, venous thromboembolism, hypertension, gestational diabetes, postpartum hemorrhage and a greater chance of assisted vaginal delivery or caesarean section^10^ . An analytical study conducted with more than 3,000 mothers in Buga, Colombia, between 2005 and 2015, showed correlations between maternal obesity, preeclampsia, eclampsia and gestational diabetes. Children of mothers with gestational diabetes and obesity were significantly heavier at birth^11^ .

There are few studies focused on macrosomia at the national level. Scientific evidence has focused on obstetric complications and the study of maternal-fetal metabolic disorders. In addition, the risk analysis of public health events in NBs has focused on low birthweight^12^ . However, the increased numbers of female obesity and macrosomia support the need for this to be considered an important indicator in prenatal care and surveillance due to its deleterious effects in the short, medium, and long term in the health of the newborn.

The purpose of this research was to analyze clinical factors and weight gain in pregnant women and their association with fetal macrosomia.

## METHODS

Analytical case-control study, nested in a defined cohort of NB in a second level institution responsible for the health care of the southwestern department of Antioquia between 2010–2017, and their mothers, who performed prenatal control in the same institution or in others from nearby municipalities. Information sources were the birth record, the Latin American Center for Perinatology (CLAP) record, and the maternal medical history.

Selection criteria were: mothers between 15 and 45 years old; prenatal control carried out in the institution or institutions of nearby municipalities; minimum 90% of the data in the CLAP file or clinical history; data on pre-pregnancy weight, or measured before week 14, one ≥ at week 36, and anthropometric data of the newborn. Mothers with multiple pregnancies, pre-pregnancy diabetes and NB with congenital diseases were excluded. Macrosomic NBs weighing ≥ 4,000 g were identified, as specific inclusion criteria for the study *case* group. The same inclusion criteria were applied for the *control* group, except for birth weight, which was defined as a weight between 3,000 and 3,999 g, considered adequate.

**Figure 1:**
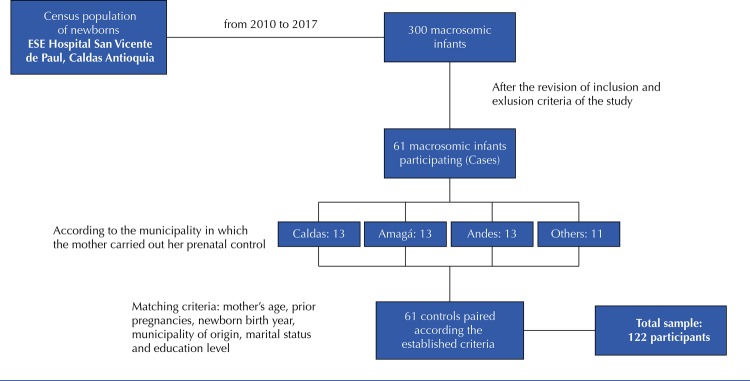
Procedure for obtaining the sample.

From the census population of NB in the institution, 300 macrosomic infants were obtained, of which 61 met the selection criteria. Pairing of cases and controls 1:1 was performed ( Figure 1 ). The following sociodemographic variables were considered according to their hierarchy: maternal age, previous pregnancies, socioeconomic level, NB year of birth, and municipality of origin. Both cases and controls had a median age of 24 years and a previous pregnancy, with prevalence of low socioeconomic levels and secondary/university education, which indicated there were no significant differences between them (p> 0.005). Marital status and educational level were considered as non-mandatory matching variables; the total sample was 61 cases and 61 controls.

Aspects such as pre-pregnancy body mass index (BMI), weight gain and pathologies during pregnancy were studied. In the neonate, data on sex, weight, length, head circumference and gestational age at birth were collected, which were classified according to data reported in the medical history, date of the last menstruation or first trimester ultrasound.

For the analysis plan, the classification of the pregnant women nutritional status according to age was considered, according to 2016 Resolution no. 2465 by the Ministry of Health and Social Protection of the Republic of Colombia^13^ . pre-pregnancy BMI in pregnant women under the age of 19 was performed using the BMI indicator for age; for those over 19 years old, classification was made according to the BMI indicator for gestational age, based on the standard proposed by Atalah et al.^14^ : underweight below 20 kg/m^2^, normal 20–24.9 kg/m^2^, overweight 25–29.9 kg/m^2^, and obesity ≥ 30 kg/m^2^. Both standards are accepted by the national standard.

The weight gain goal was calculated according to the healthy pre-pregnancy weight, equivalent to a BMI of 22.5 kg/m^2^. Weight gain in adults was classified as follows: underweight 12-18 kg, adequate weight 10–13 kg, overweight 7–10 kg, and obesity 6–7 kg^15^ . In adolescents, it was classified as: underweight between 12–20 kg, adequate weight between 12.5–17 kg, overweight between 7.5–12.5 kg and, in obese women, less than 7kg^16^ . Inadequate gain due to deficit was defined as lower than the recommended weight ranges; excessive weight gain was related to that which exceeded the recommendation, and adequate gain remained within the specified ranges.

For the classification of total weight gain, the difference between the last pre-delivery weight recorded in week 36 or later weeks and the pre-pregnancy weight recorded at week 14 or less was calculated. If the birth occurred after the week in which the last weight of the mother was recorded, weight gain was projected according to the percentile of gain of each pregnant woman. Maternal height greater than 1.55 m was considered as a cut-off point for the risk of macrosomia at birth, according to previous studies^17^ .

A systematized instrument was developed in the Epi Info program, version 7.2.1.0, for the collection of information. Collection was carried out by previously trained and standardized personnel. Descriptive analysis included absolute and relative distributions, as well as summary indicators such as arithmetic mean, standard deviation, quartiles and interquartile range. The normality criterion for some sociodemographic and clinical variables was established by the Shapiro Wilk test. U-Mann Whitney test or Student t-test for independent samples were used to determine the relationship between macrosomia and some quantifiable sociodemographic and clinical aspects. The relationship between sociodemographic aspects and clinical history with macrosomia was defined by Pearson’s chi-square test or Fisher’s exact test. Strength of association was evaluated by proportion ratio (PR), with its respective 95% confidence intervals (95%CI) (p < 0.05).

A parsimonious model was applied, which selected the variables that best explained the effect of macrosomia by a generalized linear model (GML) of Poisson regression with robust variance. Statistical data processing was performed in SPSS Software, version 23.

The research was approved by the ethics committee of the Faculty of Nursing of the University of Antioquia. Institutions in which the data collection process was carried out gave their endorsement and authorization for the review of data sources. The study was considered an “investigation with minimal risk”, with strict custody and confidentiality of information, in accordance with Statutory Law 1581, of 2012, and Resolution number 1995, of 1999.

## RESULTS

In both groups, the median age was 24 years old and 75.4% of the participants were between 19 and 34 years old; the predominant educational level (81.1%) was secondary-university; 74.6% had low socioeconomic status; 82% were affiliated with the subsidized health regime, and 68% had the presence of a partner (married or in free union).

Of the mothers, 59.8% were multiparous, 18.0% had previous abortions, and 78.7% performed six or more prenatal controls. They presented a family history of 50% arterial hypertension, and 26.2% of diabetes mellitus. Prior preeclampsia was 6.2% and, during pregnancy, 2.5%. The threat of preterm birth reached 27.8% and premature rupture of membranes, 5.7%.

The average gestational age at birth of the NB was 39 weeks in both groups, with an average birth weight in the case group of 4120 grams, and of 3334 grams in the control group. Mean length at birth was 52.3 cm and 50 cm in the case and control groups, respectively. The mean head circumference in the cases was 36 and, in the controls, 34.5 cm. The predominant sex in the NB was male, with 60.7%, compared with 39.3% of females, with a similar distribution among the groups. Regarding the type of delivery, 63% of macrosomic NBs were born by caesarean section.

The median pre-pregnancy weight of pregnant women was 60.7 Kg; 65.1 kg in the case group and 57.8 kg in control. The average height was 1.57 m. The median pre-pregnancy BMI in the studied sample was 26.6 kg/m2, and 23.2 kg/m2 in the control group. Average weight at the end of pregnancy 78.5 kg and 68.8 kg in the case and control groups, respectively. Half of the mothers started gestation with adequate weight; 28.7% overweight, 15.6% with obesity, and 5.7% underweight. The average weight gain was 12.2 kg.

Of the pregnant women who presented excess pre-pregnancy BMI, 64.8% had macrosomic children, while in pregnant women with adequate BMI the percentage was 38.2%. Macrosomia was 1.6 times more likely in pregnant women with excess pre-pregnancy BMI compared with those who did not (95%CI 1.18-2.43; p = 0.004). 63.9% were > 1.55 m tall; however, no statistical differences were found between the groups regarding this variable (p = 0.131) ( Table 1 ).

**Table 1:** Sociodemographic, anthropometric, and clinical factors associated with macrosomia.

		Total (%)	Cases n (%)	Control n (%)	p	PR (95%CI)
Age group (years)	< 19 *	19 (15.6)	8 (42.1)	11 (57.9)		
	19 to 34	92 (75.4)	47 (51.1)	45 (48.9)	0.674	1.21 (0.68–2.13)
	> 34	11 (9.0)	6 (54.5)	5 (45.5)	0.752	1.30 (0.61–2.76)
Parity	Presents prior pregnancies	73 (59.8)	37 (50.7)	36 (49.3)	0.853	1.03 (0.72–1.49)
	Does not present prior pregnancies	49 (40.2)	24 (49.0)	25 (51.0)		
Socioeconomic level	Low	91 (74.6)	45 (49.5)	46 (50.5)	0.835	0.96 (0.64–1.43)
	Medium-High	31 (25.4)	16 (51.6)	15 (48.4)		
Education level	None-Primary	23 (18.9)	11 (47.8)	12 (52.2)	0.817	1.06 (0.66–1.69)
	Secondary-University	99 (81.1)	50 (50.5)	49 (49.5)		
Maritial Status	Absence of partner	39 (32)	19 (48.7)	20 (51.3)	0.846	1.04 (0.71–1.53)
	Presence of a partner	83 (68)	42 (50.6)	41 (49.4)		
Mother height	Risk height	78 (63.9)	18 (40.9)	26 (59.1)	0.131	1.35 (0.89–2.03)
	No-risk height	44 (36.1)	43 (55.1)	35 (44.9)		
pre-pregnancy body mass index	Excess weight	54 (44.3)	35 (64.8)	19 (35.2)	0.004	1.69 (1.18–2.43)
	No excess weight	68 (55.7)	26 (38.2)	42 (61.8)		
Body Weight Gain	Inadequate due to deficit *	25 (20.5)	5 (20.0)	20 (80.0)		
	Adequate	38 (31.1)	14 (36.8)	24 (63.2)	0.179	1.84 (0.76–4.49)
	Inadequate due to excess	59 (48.4)	42 (71.2)	17 (28.8)	0.002	3.56 (1.59–7.95)
Health regimen	Subsidized/Associate	100 (82)	46 (46.0)	54 (54.0)	0.031	0.67 (0.47–0.96)
	Contributory	22 (18)	15 (68.2)	7 (31.8)		
Diabetes in pregnancy	Present	18 (14.2)	16 (88.9)	2 (11.1)	0.000	2.05 (1.56–2.71)
	Not present	104 (85.2)	45 (43.3)	59 (56.7)		
Childbirth type	Caesarean section	62 (50.8)	39 (62.9)	23 (37.1)	0.004	1.72 (1.17–2.52)
	Spontaneous	60 (49.2)	22 (36.7)	38 (63.3)		
Total of prenatal controls	Less than 6 controls	26 (21.3)	15 (57.7)	11 (42.3)	0.377	1.20 (0.81–1.78)
	6 or more controls	96 (78.7)	46 (47.9)	50 (52.1)		

PR: proportion ratio

In pregnant women with excessive weight gain, 71.2% presented macrosomic NB. The risk of macrosomia increased 3.6 times in pregnant women who had excessive weight gain, compared with those who had adequate or poor gain (95%CI 1.59–7.95; p = 0.002) ( Table 1 ).

**Figure 2:**
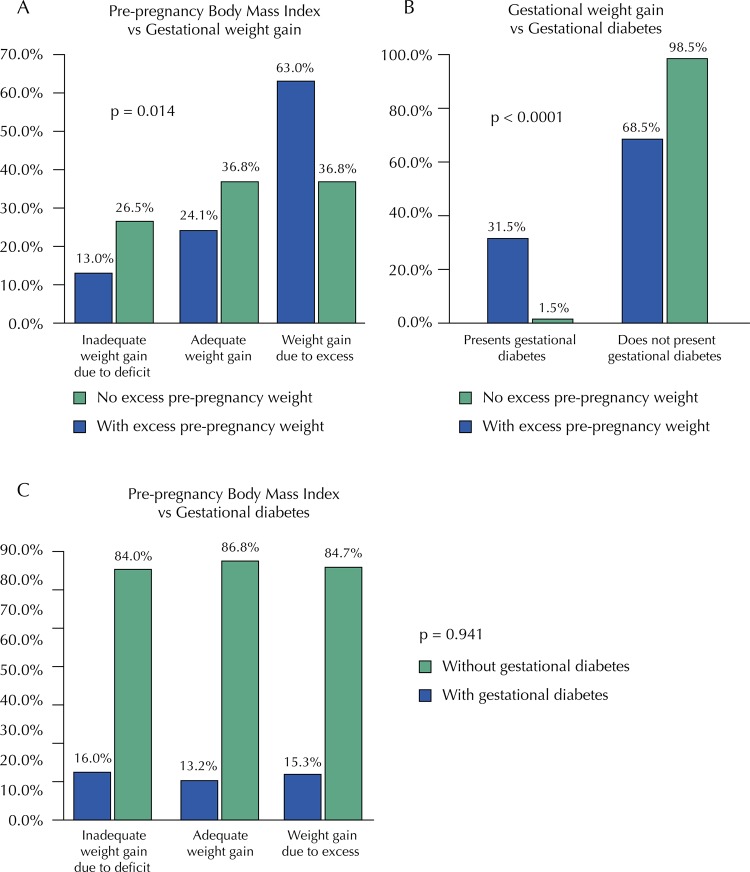
A. Pre-pregnancy Body Mass Index vs Gestational weight gain B. Gestational weight gain vs Gestational diabetes C. Pre-pregnancy Body Mass Index vs Gestational diabetes.

Of women with excess pre-pregnancy weight, 63% had excessive weight gain and 24.1% had adequate weight gain; statistically significant differences were found between pre-pregnancy BMI and weight gain classification ( Figure 2 ).

Of the pregnant women with gestational diabetes, 88.9% had macrosomic children. Macrosomia was 43.3% in those who did not have diabetes. The risk of macrosomia was twice as high in pregnant women with gestational diabetes, compared with those who did not present it (95%CI 1.56–2.71; p = 0.0001)

Of the participants with excess pre-pregnancy weight (n = 54), 31.5% had diabetes; of those who did not present pre-pregnancy excess weight (n = 68), 1.5% presented it; statistically significant differences were found between pre-pregnancy BMI and the presence of diabetes in the mother (p <0.00001). Weight gain showed no association with gestational diabetes ( Figure 2 ).

The variables that best explained macrosomia were gestational weight gain and gestational diabetes in the final multiple regression model. The probability of being macrosomic increased 3.5 times in pregnant women with excessive weight gain (95%CI 1.78–7.18) compared with pregnant women with adequate or insufficient weight gain. Likewise, pregnant women with gestational diabetes (95%CI 1.51–2.76) were twice as likely to have a macrosomic child, compared with those who did not develop this disease ( Table 2 ).

**Table 2:** Generalized linear adjustment model to compare gross values.

Group	gross p	gross PR (95%CI)	adjusted p	Adjusted PR (95%CI)
Inadequate gain due to excess	0.002	3.56 (1.59–7.95)	< 0.0001	3.58 (1.78–7.18)
Diabetes in pregnancy	< 0.0001	2.05 (1.56–271)	< 0.0001	2.04 (1.51–2.76)

PR: proportion ratio

## DISCUSSION

Maternal factors such as excess pre-pregnancy weight, excessive weight gain, and diabetes during pregnancy increase the likelihood of macrosomia in the newborn. Overweight and obesity at the start of pregnancy were risk factors for excessive weight gain and the onset of gestational diabetes.

Excess weight changes the intrauterine environment and leads to a higher risk of obstetric and neonatal complications. In this study, almost half of the pregnant women were overweight or obesity before pregnancy. This is in line with results of the 2015 National Survey of Nutrition Situation in Colombia^3^ , which found that 39.9% of them presented excess weight.

The relationship between excess pre-pregnancy weight and newborn macrosomia has been evidenced, which are conditions associated with an increased risk of perinatal mortality and neonatal morbidity. In an investigation conducted by Koyanagi et al. in 23 countries, he concluded that excess pre-pregnancy BMI is associated with a birth weight > 4000 g^7^ . Other studies conducted in countries such as Cameroon^18^ , the USA^19^ , Lebanon^20^ , Uruguay^6^ , Peru^17^ , Argentina^21^ and Paraguay^22^ showed an association between excess pre-pregnancy weight and macrosomia, similarly to this study. Excess pre-pregnancy weight has contributed to the increased prevalence of macrosomia in different countries, in some cases, regardless of weight gain in pregnancy^7^ . The evidence of a relationship between BMI and effects on birthweight is overwhelming^23^ .

Pregnant women with excess pre-pregnancy weight exceeded the gain recommendations in this study. Multiple investigations in different countries coincide with these findings and state that pre-pregnancy overweight or obesity imply a greater possibility of exceeding the recommended weight gains, which aggravates the prospect for this group of pregnant women^2 , 20 , 23 , 24^ .

Excessive gestational weight gain was the variable with the greatest effect on the probability of a newborn with macrosomia, according to the findings of this investigation. Other authors have reported similar results and state that obese and overweight women had higher proportions of total weight gain^23 , 25 , 26^ . This can generate a fetus of greater birthweight, even in women without pre-pregnancy excess weight^6 , 22^ . An expert review published in 2016 shows that women were more likely to have macrosomia when they had excessive BMI variations. This shows the need to carry out a strict monitoring of weight gain, especially in those women with pre-pregnancy excess. They require a differentiated attention that contributes to achieving a gain adjusted to their pre-pregnancy weight^27^ .

Another aspect associated with the appearance of macrosomia was gestational diabetes mellitus. Hyperglycemia states are linearly associated with the increase in newborn weight^28^ . Additionally, the prevalence of diabetes is higher in pregnant women with excess weight, compared with pregnant women with normal BMI, and grows as the BMI increases. Women with BMI > 25 are up to six times more likely to develop it and have a higher risk of simultaneous diagnoses of gestational hypertension and post-gestational diabetes^10 , 11^ .

Macrosomia occurred in a greater proportion in infants of young mothers, with a secondary or university education level, with one or without previous pregnancies, and with a low proportion of gestational diabetes. The maternal variables associated with newborn macrosomia are age > 35 years old, low educational level, greater number of children, few prenatal controls, and pre-pregnancy diabetes or that develops during pregnancy^9 , 29 , 30^ . This study indicates that pre-pregnancy excess weight and excessive weight gain have a marked effect on the onset of macrosomia, which is independent of other variables.

To restrain the spread of epidemic excess weight, women must receive a comprehensive intervention before, during, and after pregnancy. Within the strategies to improve sexual and reproductive health in Colombia, establishing a guide or protocol containing guidelines addressed to decision-makers, public health policymakers, health care institutions, and interprofessional groups at all levels of attention is a priority. These guidelines should be geared towards the prevention and timely intervention of excess weight in women, as well as the prevention of excessive gestational weight gain in pregnant women, regardless of their pre-pregnancy BMI. This may contribute to the reduction of fetal macrosomia as one of its associated complications.

Prenatal control programs and professionals responsible for the care of pregnant woman are called to take the lead in this matter. A differential and contextualized care must be devised. Interventions for pregnant women with excess weight should include: education and nutritional care, physical activity according to maternal health, empowerment of women around their prenatal care and, especially, their weight gain and dietary interventions^4^ . Interventions should contribute to weight gains adjusted to the pre-pregnancy BMI and motivate mothers to improve the selection, portion size and preparation of foods with low caloric density and higher nutritional value, favoring the prevention of micro-nutrients deficit risk and the management of anxiety face food, to achieve the proposed goals.

A limitation of this study is the collection of data from secondary sources, which may affect their quality. pre-pregnancy BMI and weight gain in pregnancy are modifiable risk factors that are susceptible to nutritional intervention and can contribute to minimizing adverse perinatal outcomes. This analysis contributes to the discussion about maternal excess weight and macrosomia in newborns, as aspects of paramount importance given the weight gain in women of childbearing age worldwide, and the deleterious effects of macrosomia in the short-, medium-, and long-term health. The results of this study are of great relevance for the department of Antioquia and the rest of the country, and they are expected to be taken as input at the national level to declare macrosomia at birth as an indicator of public health.
