# Supplementary material for: Factors associated with fetal macrosomia
Source: Rev Saude Publica. 2019 Nov 18;53:100. doi: 10.11606/s1518-8787.2019053001269 (PMC6863107; doi:10.11606/s1518-8787.2019053001269)
Supplement: Translation

# Translation: Factores asociados a la macrosomía fetal

**Authors:** Vanessa Agudelo-Espitia, Beatriz Elena Parra-Sosa, Sandra L Restrepo-Mesa

**Affiliations:** I Universidad de Antioquia. Escuela de Nutricion y Dietetica. Colombia; II Universidad de Antioquia. Escuela de Nutrición y Dietética. Grupo de Investigación Alimentación y Nutrición Humana. Magíster en Ciencias Básicas Biomédicas. Colombia; III Universidad de Antioquia. Escuela de Nutrición y Dietética. Grupo de Investigación Alimentación y Nutrición Humana. Magíster en Salud Colectiva. Colombia

**Keywords:** Macrosomía Fetal, Fatores de Riesco, Peso al Nacer, Aumento de Peso, Fenómenos Fisiológicos de la Nutrición Prenatal, Salud Materno-Infantil

## Abstract

**OBJETIVO:**

Analizar factores clínicos y ganancia de peso en gestantes y su asociación con la macrosomía fetal.

**MÉTODOS:**

Estudio de casos y controles a partir de registros secundarios de información en una institución pública en Antioquia, Colombia, entre 2010 y 2017. Se definieron como casos los recién nacidos con peso ≥ 4.000 g y como controles aquellos con peso entre 3.000 y 3.999 g. Participaron 122 gestantes, 61 casos y 61 controles. Se estableció razón de proporción para evaluar los factores asociados con la macrosomía y se realizó un modelo lineal generalizado de regresión de Poisson con varianza robusta para evaluar los aspectos que mejor explicaron la macrosomía en el neonato.

**MÉTODOS:**

Un estudio de casos y controles, utilizando registros de información secundaria. Se definieron casos utilizando un peso de recién nacido de ≥ 4000 g, mientras que los controles se definieron como el peso del recién nacido entre 3000–3999g. Se estableció una relación de proporción (RP) para evaluar los factores asociados con macrosomía y un modelo lineal generalizado (GLM) de regresión de Poisson con varianza robusta se utilizó para evaluar los aspectos que mejor explicaban la macrosomía en el neonato.

**RESULTADOS:**

Del total de participantes, 44,3% tuvieron exceso de peso pregestacional y 48,4% ganancia de peso gestacional excesiva. Se hallaron diferencias estadísticamente significativas entre los grupos en las variables índice de masa corporal pregestacional (p = 0,004), ganancia de peso gestacional (p = 0,000), diabetes gestacional (p = 0,000) y tipo de parto (p = 0,004). Según el modelo de regresión, fue 3.5 veces más probable un recién nacido macrosómico en mujeres con ganancia de peso gestacional excesiva (IC95% 1,78-7,18) y fue dos veces más probable en aquellas que presentaron diabetes gestacional (IC95% 1,51-2,76). De las mujeres con exceso de peso pregestacional, 63% tuvieron ganancia de peso excesiva.

**CONCLUSIONES:**

El índice de masa corporal pregestacional elevado, el aumento excesivo de peso en el embarazo y la presencia de diabetes gestacional se asocian con un mayor riesgo de macrosomía del neonato. Estos constituyen factores de riesgo modificables que son susceptibles de intervención nutricional, con el fin de minimizar los resultados perinatales adversos.

## INTRODUCTION

Las cifras de exceso de peso han tenido un aumento en la población mundial en las últimas décadas. Según el reporte del Panorama de Seguridad Alimentaria y Nutricional en América Latina y el Caribe se evidenció cómo el sobrepeso y la obesidad en más de 20 países del continente fue 10 puntos porcentuales mayor en mujeres que en hombres en el 2016^1^ . Un informe realizado por la Organización de las Naciones Unidas en el 2015 expone que una de cada cuatro mujeres en edad adulta es obesa^2^ . La situación no es diferente en Colombia: la prevalencia de sobrepeso y obesidad en la población adulta es 55,2% en mujeres frente a 45,6% en hombres^3^ .

Cada vez más mujeres inician la gestación en exceso de peso. La Encuesta Nacional de la Situación Nutricional en Colombia de 2015 indica que 39,9% de las gestantes de todos los grupos de edad presentaron exceso de peso (24,7% sobrepeso y 15,2% obesidad) en el país^3^ . Otros estudios observacionales en muestras representativas de gestantes en países como Perú^4^ , Brasil^5^ y Uruguay^6^ , muestran prevalencias de exceso de peso de 63,8%, 47,5% y 32,6%, respectivamente.

Este panorama afecta directamente el peso al nacer. Un estudio realizado en 23 países mostró una prevalencia de macrosomía de 4,5% y de 5,4% en América Latina. En países desarrollados, esta osciló entre 5% y 20%, y se ha reportado un aumento de 15-25% en las últimas tres décadas^7^ . En Colombia, se realizó una investigación a partir del Registro de Nacido Vivo del Departamento Administrativo de Estadísticas Vitales de los años 2002 a 2011, entre los cuales se registraron cerca de 6.000.000 nacimientos. El bajo peso al nacer fue 3,8%, mientras la macrosomía alcanzó 4,5%, en los recién nacidos (RN) a término^8^ .

El binomio exceso de peso-gestación puede marcar el origen de una gama de enfermedades. Diversos mecanismos parecen confluir en la programación metabólica y la transferencia generacional de la obesidad y sus comorbilidades asociadas: inflamación, estrés oxidativo, desórdenes neurohormonales, modificaciones epigenéticas, calidad de la microbiota materna, macrosomía y mayor adiposidad fetal^9^ ; además de mayor admisión a la unidad de cuidados intensivos neonatales, alteraciones respiratorias y muerte neonatal^10^ .

Los riesgos del exceso de peso en la madre incluyen preeclampsia, tromboembolismo venoso, hipertensión, diabetes gestacional, hemorragia posparto y mayor probabilidad de parto vaginal asistido o cesárea^10^ . Un estudio analítico realizado con más de 3.000 madres en Buga, Colombia, entre 2005 y 2015, mostró correlaciones entre obesidad materna, preeclampsia, eclampsia y diabetes gestacional. Los hijos de madres con diabetes gestacional y obesidad fueron significativamente de mayor peso al nacer^11^ .

Son escasos los estudios centrados en la macrosomía en el ámbito nacional. La evidencia científica se ha enfocado en las complicaciones obstétricas y el estudio de las alteraciones metabólicas materno-fetales. Además, el análisis de riesgo de eventos en salud pública en los RN se ha centrado en el bajo peso al nacer^12^ . Sin embargo, el incremento en las cifras de obesidad femenina y de macrosomía sustenta la necesidad de que esta sea considerada un indicador importante en la atención y vigilancia prenatal por sus efectos deletéreos a corto, mediano y largo plazo en la salud del neonato.

La presente investigación tuvo como propósito analizar factores clínicos y ganancia de peso en gestantes y su asociación con la macrosomía fetal.

## MÉTODOS

Estudio analítico de casos y controles, anidado en una cohorte definida de RN en una institución de segundo nivel referente de la atención en salud del suroeste del departamento de Antioquia entre 2010–2017, y sus madres, quienes realizaron control prenatal en la misma institución o en otras de municipios cercanos. Las fuentes de información fueron el registro de parto, la ficha del Centro Latinoamericano de Perinatología (CLAP) y la historia clínica materna.

Los criterios de selección fueron: edad de la madre entre 15 y 45 años, control prenatal en la institución o instituciones de municipios cercanos, mínimo 90% de los datos en la ficha CLAP o la historia clínica, un dato de peso pregestacional o antes de la semana 14, uno ≥ a la semana 36 y los datos antropométricos del neonato. Se excluyeron las madres con embarazos múltiples, diabetes previa a la gestación y RN con enfermedades congénitas. Se identificaron los RN macrosómicos con peso ≥ 4.000 g, como criterio de inclusión específico para el grupo de *casos* del estudio. Los mismos criterios de inclusión fueron aplicados para el grupo de *controles* , excepto el peso al nacer, el cual se definió como un peso entre 3.000 y 3.999 g, considerado como adecuado.

A partir de la población censal de RN en la institución, se obtuvieron 300 neonatos macrosómicos, de los cuales 61 cumplieron los criterios de selección. Se realizó emparejamiento de casos y controles 1:1 ( Figura 1 ). Se consideraron de acuerdo con su jerarquía las siguientes variables sociodemográficas: edad materna, gestas previas, nivel socioeconómico, año de nacimiento del RN y municipio de procedencia. Tanto los casos como los controles tuvieron una mediana de edad de 24 años y una gesta previa, predominó el nivel socioeconómico bajo y la escolaridad secundaria/universitaria, lo que indicó que no se presentaron diferencias significativas entre ellos (p > 0,005). Se consideraron el estado civil y nivel educativo como variables no obligatorias de emparejamiento; la muestra total fue de 61 casos y 61 controles.

**Figura 1:** Procedimiento para obtención de la muestra.

Se estudiaron aspectos como índice de masa corporal (IMC) pregestacional, ganancia de peso y patologías durante la gestación. En el neonato se recolectaron los datos de sexo, peso, longitud, perímetro cefálico y edad gestacional al nacimiento, la cual se clasificó según los datos reportados en la historia clínica, por fecha de la última menstruación o ecografía del primer trimestre.

Para el plan análisis, se consideró la clasificación del estado nutricional de las gestantes de acuerdo a la edad, según la Resolución 2465 de 2016 del Ministerio de Salud y Protección Social de la República de Colombia^13^ . El IMC pregestacional en la embarazada menor de 19 años se realizó por medio del indicador IMC para la edad; para las mayores de 19 años, se realizó la clasificación según el indicador IMC para la edad gestacional, basado en el estándar propuesto por Atalah et al.^14^ : enflaquecida inferior a 20 kg/m^2^, normal 20–24,9 kg/m^2^, sobrepeso 25–29,9 kg/m^2^y obesidad ≥ 30 kg/m^2^. Ambos estándares acogidos por la norma nacional.

La meta de ganancia de peso se calculó de acuerdo al peso pregestacional saludable equivalente a un IMC de 22,5 kg/m^2^. La ganancia de peso en adultas se clasifico así: enflaquecidas 12-18 kg, peso adecuado 10–13 kg, sobrepeso 7–10 kg y obesidad 6–7 kg^15^ . En adolescentes, se clasificó como: enflaquecidas entre 12–20 kg, peso adecuado entre 12,5–17 kg, sobrepeso entre 7,5–12,5 kg y en las obesas menos de 7 kg^16^ . Se definió ganancia inadecuada por déficit la inferior a los rangos de peso recomendados; ganancia excesiva de peso la que superó la recomendación; y ganancia adecuada la que se mantuvo dentro de los rangos especificados.

Para la clasificación de la ganancia de peso total, se calculó la diferencia entre el último peso previo al parto registrado en la semana 36 o semanas posteriores y el peso pregestacional registrado a la semana 14 o menos. Si el nacimiento ocurrió después de la semana en la cual se registró el último peso de la madre, se proyectó la ganancia de peso de acuerdo al percentil de ganancia de cada gestante. La estatura materna mayor a 1,55 m se consideró como punto de corte para el riesgo de macrosomía al nacer de acuerdo con estudios previos^17^ .

Se elaboró un instrumento sistematizado en el programa Epi Info versión 7.2.1.0 para la recolección de la información. La recolección estuvo a cargo de personal previamente capacitado y estandarizado. El análisis descriptivo incluyó distribuciones absolutas y relativas, e indicadores de resumen como media aritmética, desviación estándar, cuartiles y rango intercuartílico. Se estableció el criterio de normalidad para algunas variables sociodemográficas y clínicas por medio de la prueba de Shapiro Wilk. Se utilizó la prueba U- Mann Whitney o la prueba t-Student para dos muestras independientes para determinar la relación entre la macrosomía y algunos aspectos sociodemográficos y clínicos cuantificables. La relación entre los aspectos sociodemográficos y los antecedentes clínicos con la macrosomía se definió mediante la prueba Chi cuadrado de independencia de Pearson o la prueba exacta de Fisher. Se evaluó la fuerza de asociación por medio de la razón de proporción (RP) con sus respectivos intervalos del 95% de confianza (IC95%) (p < 0,05).

Se aplicó un modelo parsimonioso que seleccionó las variables que mejor explicaron el efecto de la macrosomía, por un modelo lineal generalizado (GML) de regresión de Poisson con varianza robusta. El procesamiento estadístico de datos se realizó en el Software SPSS versión 23.

La investigación fue aprobada por el comité de ética de la Facultad de Enfermería de la Universidad de Antioquia. Las instituciones en las que se realizó el proceso de recolección de datos dieron su aval y autorización para la revisión de las fuentes de datos. Se consideró una “investigación con riesgo mínimo”, con estricta custodia y confidencialidad de la información, de acuerdo con la Ley Estatutaria 1581 de 2012 y la Resolución Número 1995 de 1999.

## RESULTADOS

En ambos grupos, la mediana de edad fue 24 años y 75,4% de las participantes se encontraban entre los 19 y 34 años; el nivel educativo predominante (81,1%) fue secundaria-universitaria; 74,6% tenían nivel socioeconómico bajo; 82% estaban afiliadas al régimen subsidiado de salud; y 68% contaban con presencia de compañero (casadas o en unión libre).

El 59,8% de las madres fueron multíparas, 18,0% tuvieron abortos previos y 78,7% realizaron seis o más controles prenatales. Presentaron antecedente familiar de hipertensión arterial 50%, y 26,2% de diabetes mellitus. La preeclampsia previa fue 6,2% y durante la gestación 2,5%. La amenaza de parto pretérmino alcanzó 27,8% y la ruptura prematura de membranas 5,7%.

La edad gestacional promedio al nacimiento del RN fue 39 semanas en ambos grupos, con un promedio de peso al nacer en el grupo de casos de 4120 gramos, mientras que en los controles 3334 gramos. La media de longitud al nacimiento fue 52,3 y 50 cm en los casos y en los controles respectivamente. La media del perímetro cefálico en los casos fue 36 y en los controles 34,5 cm. El sexo predominante en los RN fue el masculino con 60,7%, frente a 39,3% del sexo femenino, con una distribución similar entre los grupos. En cuanto al tipo de parto, 63% de los RN macrosómicos nacieron por cesárea.

La mediana del peso pregestacional de las gestantes fue 60,7 Kg; 65,1 kg en los casos y 57,8 kg en los controles. La media de estatura fue 1,57 m. La mediana del IMC pregestacional en los casos fue 26,6 kg/m2 y 23,2 kg/m2 en los controles. El peso promedio al finalizar la gestación en los casos fue 78,5 kg y 68,8 kg en los controles. La mitad de las madres inició la gestación con peso adecuado; 28,7% con sobrepeso, 15,6% con obesidad y 5,7% en bajo peso. La media de ganancia de peso fue 12,2 kg.

De las gestantes que presentaron IMC pregestacional en exceso, 64,8% tuvieron niños macrosómicos, y en las gestantes con IMC adecuado 38,2% . La macrosomía fue 1,6 veces más probable en las gestantes con IMC pregestacional en exceso frente a las que no lo tuvieron (IC95% 1,18–2,43; p = 0,004). El 63,9% tuvieron estatura > 1,55 m; sin embargo, no se hallaron diferencias estadísticas entre los grupos respecto a esta variable (p = 0,131) ( Tabla 1 ).

**Tabla 1:** Factores sociodemográficos, antropométricos y clínicos asociados con la macrosomía.

		Total (%)	Casos n (%)	Controles n (%)	p	RP (IC95%)
Grupo de edad (años)	< 19 *	19 (15,6)	8 (42,1)	11 (57,9)		
	19 a 34	92 (75,4)	47 (51,1)	45 (48,9)	0,674	1,21 (0,68–2,13)
	> 34	11 (9,0)	6 (54,5)	5 (45,5)	0,752	1,30 (0,61–2,76)
Paridad	Presenta gestas previas	73 (59,8)	37 (50,7)	36 (49,3)	0,853	1,03 (0,72–1,49)
	No presenta gestas previas	49 (40,2)	24 (49,0)	25 (51,0)		
Nivel socioeconómico	Bajo	91 (74,6)	45 (49,5)	46 (50,5)	0,835	0,96 (0,64–1,43)
	Medio-Alto	31 (25,4)	16 (51,6)	15 (48,4)		
Escolaridad	Ninguno-Primaria	23 (18,9)	11 (47,8)	12 (52,2)	0,817	1,06 (0,66–1,69)
	Secundaria-Universitario	99 (81,1)	50 (50,5)	49 (49,5)		
Estado civil	Ausencia del compañero	39 (32)	19 (48,7)	20 (51,3)	0,846	1,04 (0,71–1,53)
	Presencia del compañero	83 (68)	42 (50,6)	41 (49,4)		
Talla materna	Talla de riesgo	78 (63,9)	18 (40,9)	26 (59,1)	0,131	1,35 (0,89–2,03)
	Talla de no riesgo	44 (36,1)	43 (55,1)	35 (44,9)		
Índice de masa corporal Pregestacional	Con exceso de peso	54 (44,3)	35 (64,8)	19 (35,2)	0,004	1,69 (1,18–2,43)
	Sin exceso de peso	68 (55,7)	26 (38,2)	42 (61,8)		
Ganancia de peso	Inadecuada por déficit*	25 (20,5)	5 (20,0)	20 (80,0)		
	Adecuada	38 (31,1)	14 (36,8)	24 (63,2)	0,179	1,84 (0,76–4,49)
	Inadecuada por exceso	59 (48,4)	42 (71,2)	17 (28,8)	0,002	3,56 (1,59–7,95)
Régimen de salud	Subsidiado/ Vinculado	100 (82)	46 (46,0)	54 (54,0)	0,031	0,67 (0,47–0,96)
	Contributivo	22 (18)	15 (68,2)	7 (31,8)		
Diabetes en la gestación	Presenta	18 (14,2)	16 (88,9)	2 (11,1)	0,000	2,05 (1,56–2,71)
	No presenta	104 (85,2)	45 (43,3)	59 (56,7)		
Tipo de parto	Cesárea	62 (50,8)	39 (62,9)	23 (37,1)	0,004	1,72 (1,17–2,52)
	Espontáneo	60 (49,2)	22 (36,7)	38 (63,3)		
Total controles prenatales	Menos de 6 controles	26 (21,3)	15 (57,7)	11 (42,3)	0,377	1,20 (0,81–1,78)
	6 o más controles	96 (78,7)	46 (47,9)	50 (52,1)		

RP: razón de proporción

En las gestantes con excesiva ganancia de peso, 71,2% presentaron RN macrosómicos. El riesgo de macrosomía se incrementó 3,6 veces en las gestantes que tuvieron ganancias excesivas de peso, en comparación con las que tuvieron ganancia adecuada o deficiente (IC95% 1,59–7,95; p = 0,002) ( Tabla 1 )

De las mujeres con exceso de peso pregestacional, 63% tuvieron ganancia de peso excesiva y 24,1% una ganancia de peso adecuada; se hallaron diferencias estadísticamente significativas entre el IMC pregestacional y la clasificación de la ganancia de peso ( Figura 2 ).

**Figura 2:** A. Índice de masa corporal pregestacional vs ganancia de peso gestacional B. Ganancia de peso gestacional vs Diabetes gestacional C. Índice de Masa Corporal pregestacional vs Diabetes gestacional.

De las gestantes con diabetes gestacional, 88,9% presentaron niños macrosómicos. La macrosomía fue 43,3% en aquellas que no presentaron diabetes. El riesgo de macrosomía fue dos veces mayor en gestantes con diabetes gestacional, frente a las que no la presentaron (IC95% 1,56–2,71; p = 0,0001) ( Tabla 1 ).

De las participantes con exceso de peso pregestacional (n = 54), 31,5% presentaron diabetes; de las que no presentaron exceso de peso pregestacional (n = 68), 1,5% la presentaron; se hallaron diferencias estadísticamente significativas entre el IMC pregestacional y la presencia de diabetes en la madre (p < 0,00001). La ganancia de peso no mostró asociación con la diabetes gestacional ( Figura 2 ).

Las variables que mejor explicaron la macrosomía fueron la ganancia de peso gestacional y la diabetes gestacional en el modelo final de regresión múltiple. La probabilidad de ser macrosómico incrementó 3,5 veces en las gestantes con ganancia de peso excesiva (IC95% 1,78–7,18) frente a las gestantes con ganancia de peso adecuada o insuficiente. Así mismo, fue dos veces más probable que gestantes con diabetes gestacional (IC95% 1,51–2,76) tuviesen un hijo macrosómico, frente a las que no desarrollan esta enfermedad ( Tabla 2 ).

**Tabla 2:** Modelo lineal generalizado de ajuste para comparar valores crudos.

Grupo	p crudo	RP crudo (IC95%)	p ajustado	RP ajustado (IC95%)
Ganancia inadecuada por exceso	0,002	3,56 (1,59–7,95)	< 0,0001	3,58 (1,78–7,18)
Diabetes en gestación	< 0,0001	2,05 (1,56–271)	< 0,0001	2,04 (1,51–2,76)

RP: razón de proporción

## DISCUSIÓN

Factores maternos como el exceso de peso pregestacional, la ganancia de peso excesiva y la diabetes durante la gestación, incrementan la probabilidad de macrosomía en el neonato. El sobrepeso y la obesidad al iniciar la gestación fueron factores de riesgo para la ganancia de peso excesiva y la aparición de la diabetes gestacional.

El exceso de peso cambia el medio ambiente intrauterino y conlleva a un riesgo mayor de complicaciones obstétricas y neonatales. En el presente estudio, casi la mitad de las gestantes presentó sobrepeso u obesidad pregestacional. Esto está en consonancia con los resultados de la Encuesta Nacional de la Situación Nutricional en Colombia del año 2015^3^ , en la que se encontró que 39,9% de ellas presentaron exceso de peso.

Se ha evidenciado la relación entre el exceso de peso pregestacional y la macrosomía del neonato, las cuales son condiciones asociadas a un incremento del riesgo de mortalidad perinatal y morbilidad neonatal. Una investigación realizada por Koyanagi et al. en 23 países concluyó que el IMC pregestacional en exceso se asocia con un peso al nacer > 4000 g^7^ . Otros estudios realizados en países como Camerún^18^ , EEUU^19^ , Líbano^20^ , Uruguay^6^ , Perú^17^ , Argentina^21^ y Paraguay^22^ , mostraron asociación entre el exceso de peso pregestacional y la macrosomía, tal como el presente estudio. El exceso de peso pregestacional ha contribuido al aumento de la prevalencia de macrosomía en diferentes países, en algunos casos, independientemente de la ganancia de peso en el embarazo^7^ . La evidencia es contundente en la relación entre el IMC y su efecto en el peso al nacer^23^ .

Las gestantes con exceso de peso pregestacional superaron las recomendaciones de ganancia en el presente estudio. Múltiples investigaciones en diferentes países coinciden con dichos hallazgos y exponen que el sobrepeso u obesidad pregestacional implican una mayor posibilidad de superar las ganancias de peso recomendadas, lo que agrava el panorama en este grupo de gestantes^2 , 20 , 23 , 24^ .

La ganancia excesiva de peso gestacional fue la variable de mayor efecto en la probabilidad de un recién nacido con macrosomía, según los hallazgos de la presente investigación. Otros autores han reportado resultados similares y exponen que las mujeres obesas y con sobrepeso tuvieron mayores proporciones de ganancia de peso total^23 , 25 , 26^ . Eso puede generar un feto de mayor peso al nacimiento, incluso en mujeres sin exceso de peso pregestacional^6 , 22^ . Una revisión de expertos publicada en el 2016 expone que las mujeres presentaron mayor probabilidad de macrosomia cuando tuvieron variaciones excesivas del IMC. Lo anterior muestra la necesidad de realizar una estricta vigilancia a la ganancia de peso, de manera especial en aquellas mujeres con exceso pregestacional. Ellas requieren una atención diferenciada que contribuya a alcanzar una ganancia ajustada a su peso pregestacional^27^ .

Otro aspecto asociado a la aparición de la macrosomía fue la diabetes mellitus gestacional. Los estados de hiperglicemia se asocian de manera lineal con el aumento del peso del recién nacido^28^ . Adicionalmente, la prevalencia de diabetes es mayor en las gestantes con exceso de peso, respecto a las gestantes con IMC normal, y aumenta conforme se incrementa el IMC. Las mujeres con IMC > 25 tienen hasta seis veces más probabilidades de desarrollarla y mayor riesgo de diagnósticos simultáneos de hipertensión gestacional y diabetes posterior a la gestación^10 , 11^ .

La macrosomía se presentó en mayor proporción en neonatos de madres jóvenes, con nivel educativo secundaria o universitario, con una o sin gestas previas y con baja proporción de diabetes gestacional. Las variables maternas asociadas con la macrosomía en el neonato son la edad > 35 años, nivel educativo bajo, mayor número de hijos, pocos controles prenatales y diabetes pregestacional o que se desarrolla durante la gestación^9 , 29 , 30^ . El presente estudio indica que el exceso de peso pregestacional y la ganancia de peso excesiva tienen un marcado efecto en la aparición de la macrosomía, que es independiente de otras variables.

Para contener la propagación de la epidemia del exceso de peso, es necesario que la mujer reciba una intervención integral antes, durante y después de la gestación. Dentro de las estrategias para mejorar la salud sexual y reproductiva en Colombia, es prioritario establecer una guía o protocolo que contenga las directrices dirigidas a los tomadores de decisiones, generadores de políticas en salud pública, instituciones prestadoras de salud y grupos interprofesionales en todos los niveles de atención. Esas directrices deben ser orientadas hacia la prevención e intervención oportuna del exceso de peso en la mujer y la prevención de la ganancia de peso gestacional excesiva en las gestantes independiente de su IMC pregestacional. Eso puede contribuir a la reducción de la macrosomía fetal como una de sus complicaciones asociadas.

Los programas de control prenatal y los profesionales encargados de la atención de la gestante están llamados a tomar el liderazgo de este asunto. Es necesario generar una atención diferencial y contextualizada. Las intervenciones para gestantes con exceso de peso deben incluir: educación y atención nutricional, actividad física de acuerdo con la salud materna, empoderamiento de la mujer en torno a su cuidado prenatal y, en especial, a su ganancia de peso e intervenciones dietéticas^4^ . Las Intervenciones deben contribuir a ganancias de peso ajustadas al IMC pregestacional, a motivar a las madres para mejorar la selección, el tamaño de las porciones y la preparación de los alimentos de baja densidad calórica y mayor valor nutricional, favorecer la prevención del riesgo de déficit de micronutrientes y el manejo de la ansiedad frente a la alimentación para alcanzar las metas propuestas.

Una limitación de este estudio es la toma de los datos de fuentes secundarias, que pueden incidir sobre la calidad de los mismos. El IMC pregestacional y la ganancia de peso en el embarazo constituyen factores de riesgo modificables que son susceptibles de intervención nutricional y que pueden contribuir a minimizar los resultados perinatales adversos. Este análisis contribuye a la discusión en torno al exceso de peso materno y la macrosomía en el neonato, como aspectos de trascendental importancia dado el aumento de peso en las mujeres en edad fértil en el ámbito mundial, y los efectos deletéreos de la macrosomía en la salud a corto, mediano y largo plazo. Los resultados de este estudio son de gran relevancia para el departamento de Antioquia y el resto del país, y se espera sean tomados como insumo en el ámbito nacional para declarar la macrosomía al nacer como un indicador de salud pública.
